# Experimental Models of Microvascular Immunopathology: The Example of Cerebral Malaria

**Published:** 2014-01-06

**Authors:** Fatima El-Assaad, Valery Combes, Georges ER Grau

**Affiliations:** 1 Professor Chair of Vascular Immunology, Department of Pathology, Sydney Medical School, The University of Sydney, Address: Medical Foundation Building (K25)Room 208, 92 – 94 Parramatta Rd, Sydney, NSW, 2006, Australia; 2 Vascular Immunology Unit, Department of Pathology, Sydney Medical School, Medical Foundation Building, The University of Sydney Level 2, Room 20892-94 Parramatta Rd, Sydney, NSW, 2006, Australia

**Keywords:** Malaria, Cerebral malaria, Experimental malaria, *Plasmodium* Immunopathology

## Abstract

Human cerebral malaria is a severe and often lethal complication of *Plasmodium falciparum* infection. Complex host and parasite interactions should the precise mechanisms involved in the onset of this neuropathology. Adhesion of parasitised red blood cells and host cells to endothelial cells lead to profound endothelial alterations that trigger immunopathological changes, varying degrees of brain oedema and can compromise cerebral blood flow, cause cranial nerve dysfunction and hypoxia. Study of the cerebral pathology in human patients is limited to clinical and genetic field studies in endemic areas, thus cerebral malaria (CM) research relies heavily on experimental models. The availability of malaria models allows study from the inoculation of *Plasmodium* to the onset of disease and permit invasive experiments. Here, we discuss some aspects of our current understanding of CM, the experimental models available and some important recent findings extrapolated from these models.

## Introduction

Without highly effective antimalarial treatment, death from severe *Plasmodium falciparum* (*P. falciparum*) malaria ensues just hours following admission to a hospital or clinic [1]. More than three billion individuals over a 109 malaria-endemic countries are at risk of infection, particularly children under five [2] and non-immune adults. One to two percent of individuals infected with *P. falciparum* develop severe malaria, with the most serious manifestation known as cerebral malaria (CM).

*P. falciparum* is transmitted to the human host by the female *Anopheles* mosquito causing a debilitating cycle of parasite differentiation, invasion and replication within the red blood cell (RBC). Rupture of the RBC and the subsequent release of parasites and their toxins activate immunological responses within the host, leading to the manifestation of this syndrome. This review addresses the value and application of data extrapolated from *in vitro* and in vivo models of CM to the human syndrome. We have focused on the use of human and murine microvascular endothelial cells (MVEC), retinal whole mount preparations and primate or, rodent in vivo models to discuss recently discovered parameters such as microparticles (MP) and miRNA and less on those that have been extensively reviewed previously [3–18].

## Human CM: Clinical Signs and Pathophysiological Hallmarks

Immense pressure exists to quickly identify patients presenting with CM, as they are at high risk of death following hospital admission. Intensive care monitoring and antimalarial treatment is paramount to life saving care, but in resource poor countries where the highest mortality rates exist, facilities are severely limited. Those infected face poor prognosis and clinicians have a short window period in which to treat before death ensues.

The clinical presentation of CM is influenced by the immune status of the population and their level of exposure [19,20]. Notable differences in the presentation of CM exist between children and non-immune adults [21]. Typical clinical signs of *P. falciparum* infection include fever, nausea, malaise, anorexia, headache, joint ache and delirium [22]. Coma is the most indicative sign of CM as accessed via Blantyre coma score for children and Glasgow coma score for adults [22,23]. Other signs include seizures, respiratory distress, hypoglycaemia, circulatory collapse, spontaneous bleeding, intracranial hypertension, acidosis, prostration and severe malarial anaemia [21]. Surviving patients experience neurocognitive deficits particularly with speech and movement, post-infection [24–27].

The diagnosis of CM is based on the presence of unarousable coma, confirmation of peripheral parasitaemia and the exclusion of encephalopathies of other causes [28]. Because the diagnosis is based on exclusion, CM can be incorrectly considered in the differential diagnosis of many viral encephalopathies [29].

The pathogenesis of CM is complex, and manifestations result from interplay between several factors including those that relate to transmission parameters, virulence and drug sensitivities of *P. falciparum* as well those relating to the host such as genetics, nutrition and immune status.

**Figure 1:**
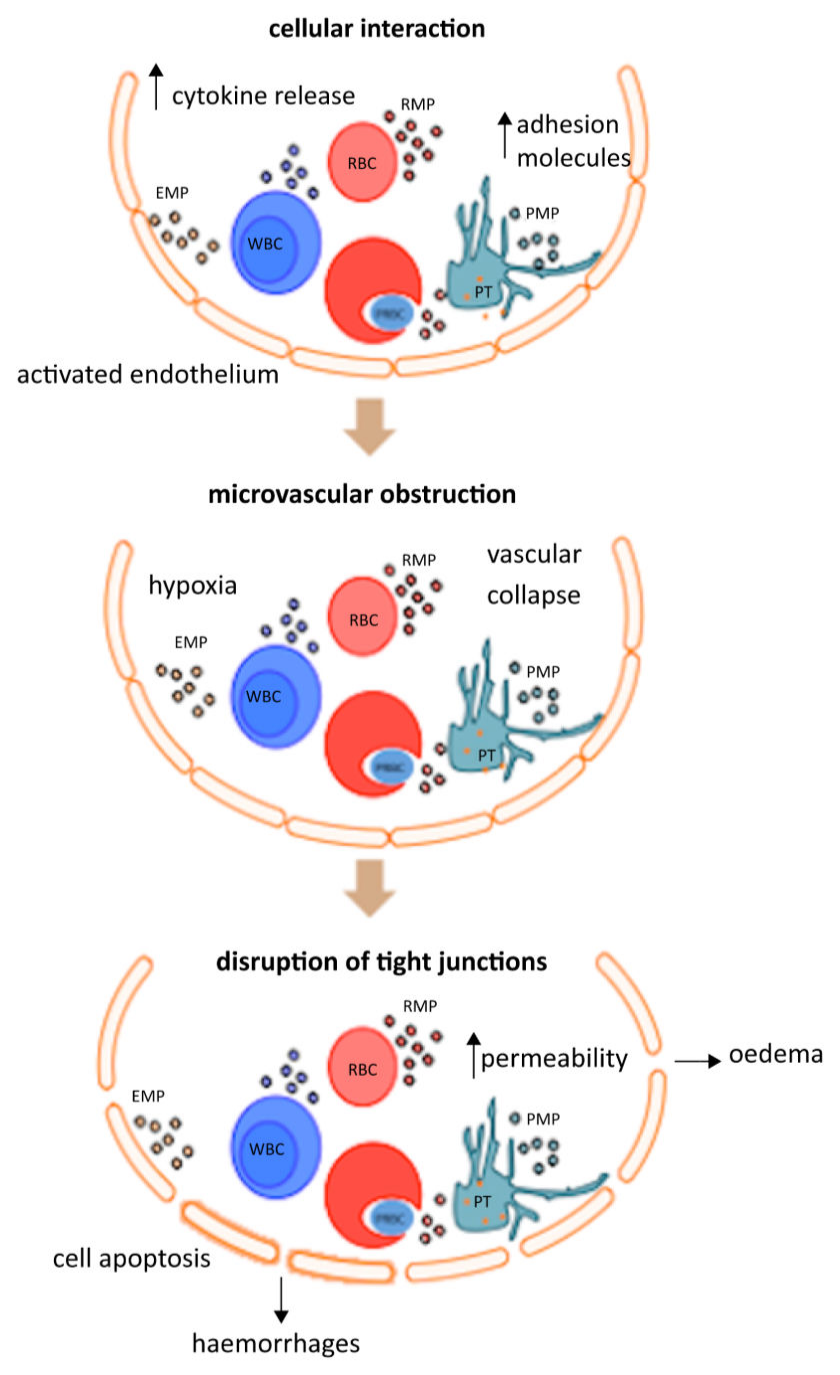
Representative diagram of the microvascular lesion underlying the development of CM. Infection with *P. falciparum* induces the production of cytokine and chemokines, activates EC lining blood vessels and upregulates cell-adhesion molecules. PRBC and host cells such as RBC, platelets and leukocytes adhere to vessel walls through the interaction of ligands and receptors. The release of MP, malarial products, toxic mediators and the activated endothelium, together facilitate the adhesive cellular interaction within the vessel lumen leading to microvascular obstruction. The arrested cells impinge on the integrity of the blood brain barrier, disrupt tight junctions, cell viability and function leading to oedema and possible haemorrhages.

**Figure 2:**
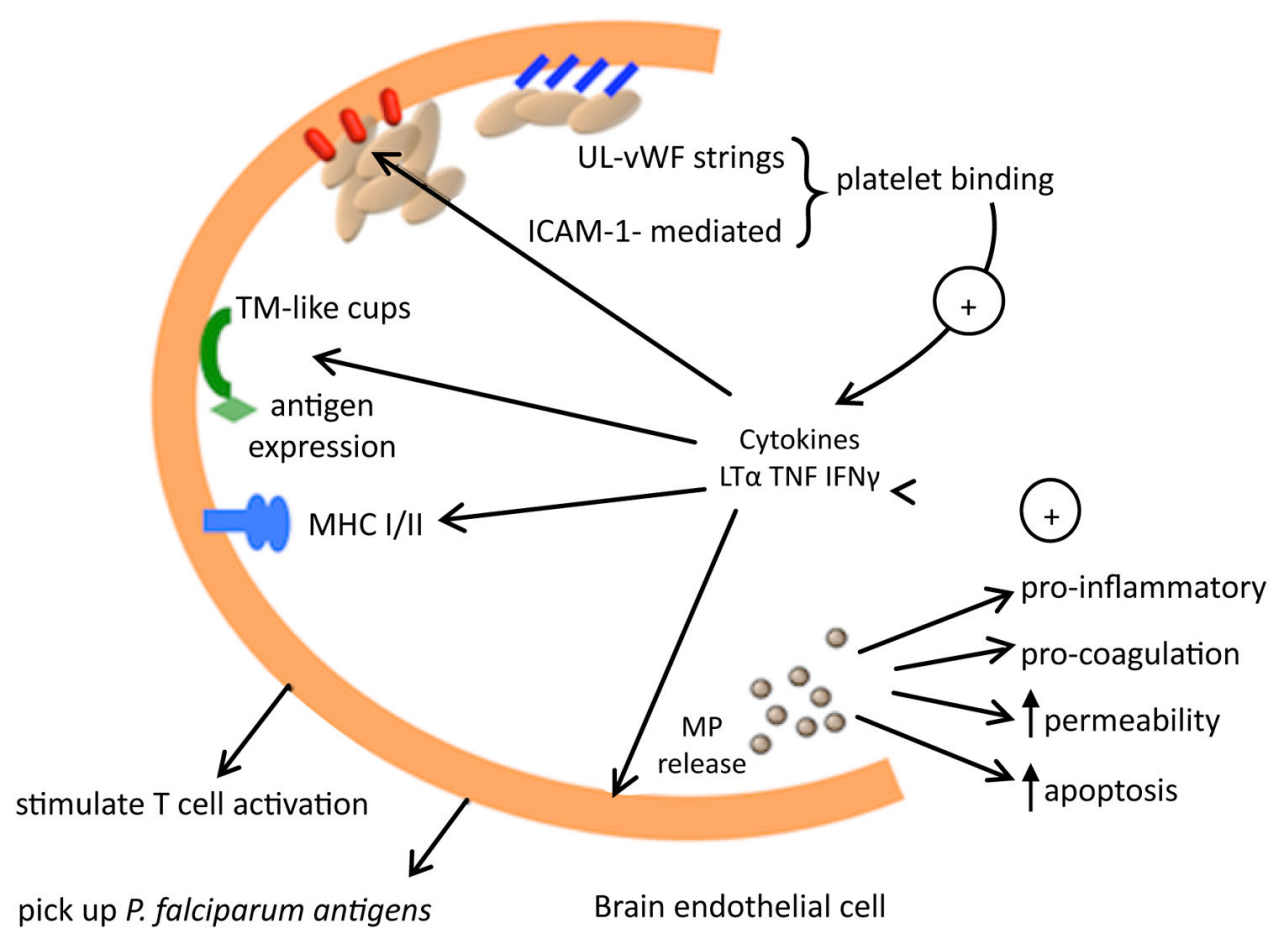
Endothelial-mediated cellular cross talks underlie immunopathological mechanisms in CM. Following upregulation of EC surface receptors and engagement with PRBC, the PRBC-EC interaction continues with a diffusion of membrane elements and the formation of tight transmigration (TM)-like cups. TNF and IFNγ are known to modulate MHC II expression shown to be related to the genetic susceptibility to CM. MHC II expression on the endothelium enables the take up of *P. falciparum* antigens supporting their role as antigen presenting cells and also supporting and stimulating T cell proliferation *in vitro*. The continued production of cytokines stimulates the release of pro-inflammatory, pro-coagulant MP from host cells. MP express antigens from their cell of origin as well as negatively charged phospholipids, triggering additional cellular interactions in the inflammatory response such as cell adhesion, permeability and cell death. MP can be found along the inner vessel wall at the site of PRBC-platelet accumulation. Platelets accumulate via von Willebrand factor (vWF) strings or adhesion receptors such as ICAM-1. Excessive cross-talk between tethered platelets, EC and their MP progeny induces alteration of the BBB integrity, promotes the secretion of cytokines and increases the adhesiveness which in turn fuels the exacerbating cycle.

This neurovascular pathology is characterised by the sequestration of mature parasitised and non-parasitised red blood cells (PRBC and NRBC) and other cell types such as platelets and monocytes deep within the cerebral microvasculature (Figure 1) [30,31]. Ischemia due to the irreversible endothelial damage caused by the intense congestion of the vessels is believed to account for the presence of petechial and ring haemorrhages in the brain of infected patients [32]. Widespread endothelial damage by cytokines and parasite toxins result in peri-vascular oedema parenchymal necrosis, and neuronal cell death (Figure 2) [33,34].

## Human CM: Limitations in Scope of Studies

To delineate the pathogenesis of CM, the timing and sequence of events leading to the manifestation must be known. For obvious reasons, human studies are limited to clinical and genetic field studies in endemic areas, and study of tissue at end-point, including comparative histopathology. These studies of HCM provide valuable descriptive and correlative data. Although to demonstrate causality and the underlying immunopathogenesis, invasive and interventional studies, which of course are unethical, would be required.

Current imaging technologies such as magnetic resonance imaging (MRI), spectroscopy (MRS) and computational tomography (CT) provide a way forward for clinicians to follow the progression of the disease [35,36]. Focal parenchymal abnormalities, haemorrhages or diffuse cerebral oedema attributed to CM can be detected and facilitate early diagnosis. However, these imaging technologies are expensive and their availability in endemic clinical settings is limited [22,37]. Ophthalmoscopy is less expensive alternative diagnostic tool used to detect malarial retinopathy in patients [38]. It is yet to be widely used but has great promise in increasing the precision of the clinical diagnosis of CM [38].

The limitations of human studies mandate the use of experimental models to supplement human data. Access to clinical samples is limited for ethical reasons contributing to the difficulty in validating data obtained from experimental models. Complementary methods of *in vivo* and *in vitro* experimental models as well as human studies are required to elucidate the mechanisms of pathophysiology in CM.

## Models Available to Study CM

In addition to clinical field and ex vivo studies (post-mortem sampling) other approaches to studying CM include, *in vitro*(modelling of CM lesions) and *in vivo* (animal models of CM) models [39]. These model systems are empowering tools that permit the study of the sequence of events from the early stages of infection and provide a platform for the testing of novel hypotheses. They have facilitated drug testing and vaccine development as well as research on mechanisms of host defence and pathogenesis of CM [40]. Here, we have chosen to address the *in vitro* and *in vivo* experimental model contributions to our understanding of HCM, specifically regarding immunopathogenesis.

The disparity between the results extrapolated from ECM and their translation to HCM have been a matter of debate. Here we aim to address the value and application of findings observed in models of CM to the paediatric syndrome. The use of ECM is justified by the number of similarities shared with HCM [5,16]. As reviewed by Hunt and Grau in 2003, the neurological signs such as convulsions, paralysis and seizures are similar [8]. A number of key histopathological features such as vascular obstruction, parenchymal haemorrhage, leukocyte adhesion, oedema and focal demyelination are observed in ECM and in HCM [8], with PRBC accumulation substantially less marked in ECM. However, several groups have reported evidence for PRBC sequestration in ECM [41–46]. With simplified coma and behavioural scoring for ECM, similar to that routinely used to assess HCM, correlations between CM signs and cerebral changes can be made quickly [47,48].

However, the value of murine models, including the PbA model, in contributing to our current understanding of the pathogenesis of CM and therapeutic strategies has been questioned [49]. The variable incidence of PRBC sequestration, the inability of mice to be infected by *P. falciparum*, and the parasitological differences between human and mouse *Plasmodium* support this idea.

### *In vitro* and *ex vivo* models

#### Human and mouse brain microvascular endothelial cells

*In vitro* models simulate the interaction between the endothelium and target cells such as RBC, platelets and MP found in the CM lesion. The endothelium of the blood brain barrier (BBB) forms the actual site of the CM lesion. Using endothelial cell lines isolated from human, mice and non-human primates, researchers have represented this interaction in both static and flow assays. MVEC have been isolated from many tissues [50], with brain and lung often used to model CM lesions, since these organs develop distinct pathology during infection. Although not a microvascular cell type, many studies have also used human umbilical cord endothelial cells in co-cultures both in static and shear-stress assays [51]. Static assays are technically simple and involve the incubation of target cells and/or parasites on subconfluent monolayers of endothelium [52]. Following incubation, non-adherent cells are washed off and adherent cells are enumerated. These static assays have contributed to our knowledge on cytoadherence although they do exclude the effect of shear forces found in blood vessels *in vivo*. Alternatively, flow based assays use laminar flow conditions simulating the physiological shear stress of blood vessels. These assays enable the quantitation of adherent cells, and allow close study of the receptors and ligands mediating the binding, whilst withstanding similar conditions experienced *in vivo* [52]. These assays can be adapted to study cytoadherence on brain, lung and placenta.

#### Retinal wholemount preparation

Retinopathy is an important clinical sign in adult [53,54] and paediatric CM [55]. Retinal pathology was first described in ECM [56], and can be detected by ophthalmoscopic examination of CM patients [38]. Recent studies describe damage of the optic and trigeminal nerve in ECM, explaining the manifestation of impaired visual acuity and neurological signs [57,58]. The retinal and cerebral vasculatures are so similar, that the retinal wholemount preparation provides information on the interaction of cells within an intact vascular plexus. This model allows the detection of microvascular changes such as haemorrhages, congestion and breakdown of BBB in the early stages of murine CM, including damage to the microglia and astrocytes [56,59].

### *In vivo* models of cerebral malaria

Available animal models of CM provide a closer approximation to the human response. Although they do not reproduce HCM entirely, the key cells that are involved in the pathogenesis of HCM, experimental CM (MCM) and the immune response pathways are similar. In addition, the clinical signs and cerebral pathology, including the lesions are comparable. The most commonly used models of CM have been developed in primates and in rodents [4,39]. The number of models available to study CM pathogenesis is limited in comparison to those available to study non-severe malaria.

#### Primates

The importance of studying *Plasmodium* infection in primates has been heightened due to findings of *P. falciparum* in wild apes once thought as infecting humans only [60]. The cross transmission of *Plasmodium* between humans and primates questions host specificity and invasion strategies of the parasite. Little evidence exists to support zoonotic transmissions, although proposals into understanding the extent of the potential are underway [61,62]. Emerging evidence for *P. knowlesi* infection in human, potentially fatal malaria, has also supported the transfer of host from primate to human [63].

Previous attempts to produce a model that used the same causative agent as in HCM were modest. Infection of the squirrel monkey *Saimiri sciureus* with *P. falciparum*, developed neurological signs with a fatal outcome, although the model lacked predictability, with variable incidence of CM [64]. Other primate models of CM include infection of the rhesus monkey (*Macaca mulatta*) with *P. coatneyi* [65], *P. knowlesi* [66] or *P. fragile* [67], whereby infected hosts develop coma and become moribund. Post-mortem examination shows characteristic vascular congestion, notably PRBC sequestration within the brain microvessels. The overproduction of cytokines (TNF, IFN and IL-1β) and the upregulation of adhesion molecules (CD36, thrombospondin and ICAM-1) are seen in these models, mimicking pathological hallmarks of the human syndrome [68]. Although contributing to knowledge on some aspects of CM, primate model use is limited by their high cost, ethical restrictions and lack of genetically modified animals available.

#### Rodents

Mouse models of CM are the most commonly used *in vivo* experimental model [39]. Several mouse models exist in which infection is induced with *Plasmodium* infected RBC including *P. yoelii* 17XL [69], PbA [70–72] *P. berghei* NK65 [73] and PbK173 [74]. Each model exhibits specific characteristics, *P. yoelii* for instance infects preferentially reticulocytes while the others infect mature RBC. PbA mouse model has many advantages that not only include the availability of genetically susceptible and resistant mouse strains, but also the ability to reproduce clinically evident neurological signs within a precise time course. Infection of CM-susceptible mice (CBA and C57BL/6) with *P. berghei* ANKA (PbA) results in reproducible fatal CM, 7 to 14 days later. PbA infection of CM-resistant mice, such as the DBA/2 and BALB/c strains, results in NCM, with mice succumbing to hyperparasitaemia and severe anaemia 3 weeks later. The availability of KO strains and the relatively inexpensive breeding and housing of the mice is also an advantage. For practical and ethical reasons rodent models are commonly used to study CM.

## The Contribution of Experimental Models to our Understanding of CM Pathophysiology

### PRBC sequestration and cytoadherence

Sequestration of *P. falciparum*-infected RBC in the microvasculature, particularly small capillaries and post-capillary venules, is a signature of HCM [75–77]. *P. falciparum* schizonts, trophozoites as well as mature gametocytes tether and roll to adhere to host receptors. By adhering to the endothelium, RBC and parasites are removed from the circulation and consequently escape clearance by the spleen, effectively implementing their survival strategy. Analysis of post-mortem tissue from individuals who died of CM show parasite sequestration within the brain, lung, spleen, intestine, skin, fat tissue and muscle [78].

During *P. falciparum* infection the ability of PRBC and NRBC to squeeze through capillaries is impaired. The sequestration of the PRBC decreases the luminal diameter and the ability of erythrocytes to deform becomes very crucial to the capillary flow rate. RBC that become more rigid, plug capillary lumens already obstructed with sequestered PRBC. The growth of the parasite within the host erythrocytes modifies its shape by increasing the internal viscosity of the cell and exerts oxidative stress on the cell through release of by-products such as lactic acid. Coupled with rosetting, this obstruction leads to decreased tissue perfusion and localised hypoxia that continues the onslaught of the endothelium.

Knob proteins provide a point of attachment and have been long known to mediate parasite sequestration in *P. falciparum* malaria [79]. Although evidence does exist for knobless cytoadherence [80–82], suggesting other mechanisms may also contribute to this phenomenon. The absence of knobs does not necessarily diminish their ability to cytoadhere [80] validating the study of mouse PRBC, that do not display the knobbly phenotype.

The mousemodels of CM have received criticism for their inability to clearly demonstrate the sequestration phenomenon in the brain as seen in HCM. One study with *P. berghei* ANKA infection in (BALB/c× C57BL/6)F1 mice, has clearly shown morphological evidence of PRBC and leucocyte sequestration in the brain, in a pattern similar to what is observed in HCM [41]. The accumulation of PRBC was also observed in the brain of PbA infected C57BL/6 mice [44]. Technically, detecting *P. berghei* sequestration *in vivo* is challenging. *P. berghei* infections are usually in asynchronous parasite stages, some stages of the cycle are indistinguishable including multiple parasite infections of a single RBC incorrectly identified as a schizont [9,43]. This hampers their detection in routine peripheral blood smears and in tissue histology. Schizonts are absent from peripheral blood smears of infected mice and only ring stages, trophozoites and gametocytes can be detected. Even technical aspects such as perfusion may also shroud the real quantitation of PRBC sequestration [44]. Also, PRBC sequestration in ECM is relatively short-lived, occurring in the final 20 hours [42], and most studies are limited by ethical constraints preventing death as the end-point.

*In vitro* assays using mouse brain MVEC have provided direct *in vitro* evidence for cytoadherence of RBC from *P. berghei* ANKA (PbA) and *P. berghei* K173 (PbK173)-infected mice on brain endothelial cells [83]. PRBC infected with *P. yoelii* 17XL also adhere to mouse brain MVEC [70].

From these assays the interaction of PRBC, monocytes, platelets and the CD36 receptor on MVEC [84,85] as well as the ICAM-1 [85], VCAM-1 and E-selectin receptors [86] has been described. Flow assays have confirmed the complexity of cytoadherence with a number of cell-adhesion molecules and selectins being implicated in the process of tethering, rolling and firm adhesion [87,88]. Understanding the variations in binding avidity to host ligands [89] and the pathways involved in the reversal [90] or inhibition of binding is important in finding new therapeutic strategies.

The site where the vascular endothelial cells are isolated from affects the gene expression and responsiveness to *Plasmodium*, thus it is important to hone in on relevant organ endothelium [77,91,92]. For example, differences exist in the mediation of cytoadherence between PRBC and the endothelium of the brain, lung and placenta. Specifically, it is known that the sequestration of PRBC in the placenta is mediated by variant surface antigens (VSA) specific for CSA, in mice and human pregnancy-related malaria [91,93]. Although, recent studies using flow cytometry-based adhesion assays, have revealed parasite adhesions other than VSA specific for CSA may also be involved [94]. Despite rodent parasites not expressing PfEMP1 homologs, mouse models of placental malaria are still informative.

The association of sequestration and clinical presentation is clear in HCM, although the causal relationship is not. Despite mouse models being criticised for not clearly demonstrating parasite sequestration, no evidence exists to suggest that the severity of HCM is related to the degree of cytoadherence [95,96].

With advancement in luciferase-based live imaging technology, evidence exists for the sequestration of PbA schizonts in the spleen, lung and adipose tissue [97], supporting the observation of organ entrapment of rodent parasites P. chabaudi chabaudi, *P. vinckei pettier, P. yoelii* schizonts [43,98]. The real-time *in vivo* imaging of these transgenic bioluminescent *P. berghei* parasites has enabled insight into the sequestration phenomenon in mice [97,99] as well as sporozoite interactions with host immune cells within the liver [100,101].

Parasite ligands and host endothelial receptors directly mediating sequestration of *P. falciparum* proteinaceous knobs on the outer membrane of RBC, erythrocyte membrane protein 1(EMP1) encoded by *P. falciparum* enable PRBC to bind multiple receptors such as CD36, intracellular adhesion molecule 1(ICAM-1), vascular adhesion molecule 1(VCAM-1), chondroitin sulphate A(CSA) and endothelial-cell selectin (E-Selectin) [102,103]. In the mouse model, CD36 has been identified as the major receptor for schizont sequestration in lung and spleen [9,97,99]. Although no CD36 mediated schizont sequestration is observed in mouse brain, evidence exists for the close association of PRBC to the brain endothelium, closely resembling what is seen in HCM [41]. It is still unclear what mediates the sequestration of parasites in the mouse brain, although it has been shown that CD36 expressing platelets, monocytes and macrophages may facilitate the cytoadherence by serving as a bridge between the parasite and endothelium [104,105]. No clear association between the binding of CD36 and PRBC and the severity of disease exist [96,106–108]. Only sparse detection of CD36 in cerebral vessels exist, with no evidence for up regulation in human cerebral malaria [109].

Endothelial protein biomarkers such as angiopoietin-1 and 2, soluble Tie-2, von Willebrand factor (vWF), vascular endothelial growth factor (VEGF), soluble ICAM-1 (sICAM-1) are predictors of paediatric CM. More specifically, higher levels of vWF and vWF pro-peptide indicate acute endothelial cell activation in patients with malaria [110]. We now know that vWF is capable of mediating the adhesion of PRBC to the endothelium via platelet decorated ultra-large vWF strings [51]. *In vitro* flow assays using *P. falciparum* PRBC and human umbilical vein endothelial cells demonstrated this adhesionvia CD36-dependent platelet bridging and reversal was achieved by treatment with vWF proteases [51].

ICAM-1 is a major adhesion molecule on the endothelium that has been described to participate in the adhesion of PRBC to the endothelium. Upregulation of ICAM-1 expression has been observed in cerebral vessels of patients who died with CM [109]. ICAM-1-mediated sequestration of PRBC in HCM has been shown [108] and parasites isolated from children with CM showed the highest binding to ICAM-1 under flow conditions [107]. This has been confirmed on brain MVECs from patients with CM, *in vitro* [77]. Contact of PRBC with the endothelium mediated partially by ICAM-1, induces diffusion of malaria antigens from parasite to the endothelium and transmigration-like cup formation, that progressively covers and engulfs the PRBC [111]. In addition to ICAM-1, PRBC form interactions with receptors CD36, P-selectin and VCAM-1 to varying degrees of strength under flow conditions [87].

Several reports of focal leucocyte recruitment in HCM [112,113] quell reports that murine models over represent the role of leucocytes in CM. Accumulation of monocytes [48,114], T cells and platelets in brain venules of PbA infected mice parallels what is observed in paediatric HCM [115] as well as adult CM [114]. Endothelial co-cultures with *P. falciparum* demonstrate the potential for leucocyte recruitment [77,116,117].

Total parasite burden is a better indicator of disease severity. Parasite biomass accumulation within the tissue microvasculature is strongly associated with the onset of ECM [44,46] and HCM [118]. Higher parasite load is found in the brain of PbA compared to PbK173-infected mice [119]. CM-susceptible mice infected with PbK173, do not develop CM, but succumb to hyper-parasitaemia and anaemia approximately 14 days post-infection [120,121]. This parasite is used to study murine non-cerebral malaria (NCM), and resembles the pulmonary pathology seen in humans, particularly with malaria-associated ARDS [122]. The mechanism by which the parasite contributes to the pathogenesis is not straight forward. Although, it’s postulated that the parasite is responsible for the direct obstruction of microvessels following sequestration in target organs and cause anaemia through their invasion of RBC.

#### Cerebral oedema

Although coma in HCM is not caused by cerebral oedema [76], brain swelling and herniation still remain characteristic of CM [123,124]. In ECM, MRI studies revealed the characteristic brain oedema along with parenchymal lesions, BBB breakdown and arterial flow perturbations [125]. This oedema contributes to the compression of cerebral arteries and nerves, perturbing the blood flow and causing cranial nerve dysfunction [57]. Histologically, enlarged perivascular spaces (PVS) are signs of brain oedema that are significantly more numerous in mice with CM [126]. Interestingly, PVS are associated with an overexpression of aquaporin-4 (AQP4) on astrocytic foot processes particularly in infected mice showing signs of neurological involvement [126] but a recent study using AQP4 KO mice suggests that this molecule does not have a role in CM pathogenesis and might be protective [127]. In HCM, although not significant, a trend towards higher AQP4 was found in Vietnamese adult patients with CM [76]. This trend may be significant if studied in paediatric CM, since it resembles ECM more closely.

#### Hypoxia

In murine CM vascular obstruction impedes blood flow leading to ischemia, and consequently contributing to the pathogenesis of CM [128]. Intravital microscopy has revealed that ECM is associated with microvascular dysfunction, vascular collapse and decreased blood flow [129]. Interestingly, hypoxic foci are more pronounced in murine CM compared to NCM and reversal of tissue hypoxia can be achieved by injection of the hypoxia-responsive hormone, erythropoietin (EPO) [130]. EPO stimulates nitric oxide (NO) production improving tissue perfusion and oxygenation *in vivo* [131]. It is well established that NO in CM, modulates endothelial activation, decreases the expression of endothelial adhesion molecules and inhibits monocyte, platelet and PRBC adhesion [132–134]. Inducible synthesis of NO by the mosquito limits parasite growth [135], although direct treatment with NO does not reduce parasite growth, even at saturable levels [136]. In ECM, NO is not required for the development of the cerebral syndrome [137] and exogenous NO is protective [134]. Nitric oxide treatment reduces ICAM-1, P-selectin in the brain of PBA infected mice, and maintains the integrity of the BBB [134]. However, low bioavailability of NO contributes to the development of the syndrome in ECM [138]. In HCM, data support a deleterious role of NO [139] as well as protective [140]. Review of current data on NO has supported a hypothesis that supplemental inhaled nitric oxide (iNO) is a solid candidate for the adjunct treatment of CM [141].

## Molecules and Cells Involved in CM Pathogenesis

### Cytokines and chemokines

Post infection, the susceptible host undergoes a Th1 response whereby over expression of TNF, lymphotoxin (LT) and IFN exacerbates the progression to CM [142]. The overproduction of TNF and it deleterious role in CM was described in ECM and confirmed in HCM [143–145]. TNF induces alterations in the endothelium [146], mediates the upregulation of adhesion molecules, induces apoptosis [147] and the further release of cytokines and chemokines and also triggers the reorganisation of plasma membrane [148]. In human clinical trials, although patients did develop CM anti-TNF monoclonal antibody attenuated the malaria fever [149]. In C57BL/6 mice, TNF deficiency does not confer protection from CM [150] suggesting other mediators may also be contributing to the syndrome.

Since the first report of raised serum levels of LT in human CM [151], studies have shown that LT and not TNF is the principle mediator of murine CM [150]. LT deficiency in C57BL/6 mice conferred complete protection against CM [150]. Produced only by lymphocytes, LT and TNF are thought to work synergistically to induce hypoglycaemia and increased serum levels of interleukin-6 in severe malaria [151]. These overlapping functions of LT and TNF indicate that several mediators are responsible for the development of CM.

Early IFN production correlates with protection from CM and this early peak is absent in mice with CM [152]. However, late production of IFN is essential for the development of the syndrome [153]. IFN mice on CM-susceptible backgrounds are resistant to CM [154,155]. IFN influences T cell effector function and it is essential for CD4+ T cell activation, which may underlie the resistance of IFN mice to CM [156]. Incubation with IFN, increases the expression of TNF receptors [157] and the synergistic relationship between TNF and IFN has been demonstrated [158]. Recent studies have shown that neutralisation of IFNβ increased survival, reduced ICAM-1 and T-cell infiltrates in the brain [159].

Murine models of CM have allowed investigations into host-dependent mechanisms. Different mouse strains exhibit different sensitivities to *Plasmodium* infection and there is increasing evidence for genetic basis for susceptibility to CM [160] and the sensitivities of brain endothelium to cytokines [77,161]. PbA infection in CM-susceptible and resistant mouse strains, allow comparison in their differential responsiveness. Clinical resistance to malaria is associated with low production capacity of IFN [162]. Brain MVECs isolated from CM-resistant and sensitive mice exhibit different sensitivities to TNF [161] as did brain EC derived from patients with CM and those with uncomplicated CM [77]. Gene analysis studies of CM-susceptible and -resistant mouse strains revealed several molecular differences in early infection [163]. Variation in host gene expression may ultimately be responsible for susceptibility to infection, influencing the clinical course of malaria. Infection with PbA significantly alters the expression of genes involved in metabolic energy pathways at the time of CM [164]. This is consistent with observations of brain metabolic perturbations by way of hypoxia, hypoglycaemia and inadequate use of oxygen observed in HCM.

#### Platelets

*In vivo* and *in vitro* studies have supported a pathogenic role for platelets in CM [164–168]. Post-mortem sampling of brain from patients with CM showed greater accumulation of platelets and a higher proportion of vessels with platelets than brain from patients with severe malarial anaemia or non-malarial encephalopathies [169]. Close study of the CM lesion using platelet-EC assays show platelets binding to and activating the endothelium and engaging in the release and transfer of substances that may exacerbate the development of the syndrome [170]. The interaction between platelets and the endothelium dramatically modulates gene expression *in vitro* on human EC [171] particularly of cytokine-, chemokine-, TGFβ-, death-receptor-, apoptosis-, erythropoietin-and TREM1-signaling pathways and potentiates cytotoxic functional changes on activated endothelium [166]. In addition, PRBC interact with CD36 expressed on platelets [172] and in synergy with TNF to induce transcriptional changes in EC [171]. Other platelet receptors gC1qR/HABP1/p32 [108] and CD31 [173] have also been identified as supporting the interaction between platelets, PRBC and endothelial cells. Imaging using a platelet-specific contrast agent revealed cytokine mediated differential platelet binding to the endothelium at earlier stages of the disease than once described using MRI [174]. As reviewed previously, platelets act as bridges between the endothelium, fibrinogen and PRBC, secreting mediators and exerting their effects at the interface of the BBB [86]. As described later in section 7, even platelet-derived microparticles enhanced *P. falciparum*-infected erythrocytes binding and altered EC functions similar to their parent cell [175].

#### T cells

Other host cells such as monocytes and T cells are active players in the pathogenesis of CM. The importance of the role of monocytes in CM pathogenesis has been reviewed previously [176]. Both CD4+ and CD8+ T cells have been implicated in HCM and ECM pathogenesis [177–179] and athymic nude mice do not develop CM [179]. Helper T lymphocytes are also involved in the pathogenesis of CM, contributing to the development of cerebral lesions with microvessel plugging and haemorrhages [70,179]. Depletion of CD4+ and CD8+ T cells reduced parasite biomass, and mice did not develop CM [44]. The concomitant presence of T cells and PRBC in the brain is critical for sequestration and the onset of CM [42,45]. CD8+ T cells accumulate in the liver in ECM, although their contribution to the progression of disease is most prominent in the brain [180]. Another line of evidence supporting this is that CD8+ T cells but no PRBC were found sequestered in the brains of mice infected with a non-CM causing parasite [42]. This was also supported in data from CM-resistant mice, where lower accumulation of PRBC and no sequestration of CD8+ T cells was observed in BALB/c mice [42]. Early evidence for leukocyte involvement in HCM [181] was later confirmed in the brains of patients who had died of CM [106,182,183].

#### MicroRNA

The host resistance and response to malaria is complex. Mouse models have allowed some insight into what influences susceptibility and the exploration of genes that may provide resistance to infection [11]. They have also enabled closer study of gene regulatory molecules involved in CM pathogenesis [118]. MicroRNA (miR)-150, miR-127 and let-7i, implicated in the innate immune response, apoptosis and monocytic proliferation were modulated in ECM but not in NCM [118]. Although CBA and C57BL/6 mice develop CM, they display differences in their expression of miRNA post-infection [118]. This suggests that strain differences may be a factor in gene expression regulation and this may have implications for the study of HCM, particularly with respect to differences in genetic makeup.

## MP as Modulators of CM

Extracellular vesicles such as exosomes and MP have now been described as having roles in cell-cell interactions, antigen presentation and immune modulation in malarial infections [184–187]. However, so far most of studies have been made on the role MP can have as biomarkers and in the development of the disease via their interactions with target cells. Exosomes are 30–100 nm membrane vesicles derived from the endosome that carry parasite proteins and are able to elicit protective immune responses in ECM [184]. MP are larger membrane vesicles (100 nm to 1 μm) produced following cell activation and apoptosis due to cell membrane remodelling and loss of phospholipid asymmetry. During vesiculation, structural proteins are distorted, phospholipids are reorganised, and specifically through the ‘flip flop’ phenomenon phosphatidylserine (PS) migrates from the inner leaflet to the outer leaflet. The role of MP in the pathogenesis of CM has been elucidated by clinical findings and supported by *in vivo* and *in vitro* studies [185–187]. Normal baseline levels of MP are present in healthy individuals although elevated levels of circulating endothelial MP have been detected in the plasma of Malawian children with acute *P. falciparum* malaria [115]. Elevated levels of platelet, RBC and leucocyte-derived have been observed in patients with *P. vivax* malaria [188]. In Cameroonian patients, elevated levels of MP were detected from EC, monocytes, platelets and RBC in adult patients with CM [189]. Platelets released the highest levels of MP and this correlated with depth of coma and thrombocytopenia in *P. falciparum*-infected patients [190] and the length of acute illness and the presence of fever in *P. vivax* patients [188]. This finding is consistent with the presence of platelet accumulation in cerebral microvessels in HCM and ECM [105,190]. Interestingly, PMP transferred platelet antigen to PRBC and not to normal RBC and increased the PRBC cytoadherence to the endothelium [175]. Uptake of PMP by the endothelium induced changes in EC phenotypes [117,175]. Plasma concentrations of RBC derived MP were highest in patients infected with *P. falciparum*, compared with *P. vivax* and *P. malariae* infected and healthy patients [191]. This supports a role for MP in the exacerbation of disease.

Data collected in human studies have also been confirmed in murine CM and NCM. Interestingly, low baseline circulating MP levels were detected in non-infected mice, and subsequently raised upon CM onset [172]. Furthermore, phenotypic analyses showed that circulating MP were predominantly from platelet, endothelial and RBC origin. Mice with NCM displayed different production patterns of MP, distinct to CM mice, again supporting a role for MP in the cerebral syndrome.

TNF is an inducer of MP production *in vitro* [192], thus the associated elevated levels of MP during infection may be related to TNF overproduction [145]. Purified MP from mice with CM displayed significantly enhanced pro-coagulant and pro-inflammatory properties compared to healthy mice [172]. In addition these MP have adhesive properties, suggesting a potential pathogenic role *in vivo* [172].

Functional loss of ATP-binding cassette A-1 (ABCA-1) on the plasma membrane disables the cells ability to respond to agonists thus decreased the number of circulating MP and conferring protection against CM [172]. Prevention of CM by pantethine treatment in PbA infected mice also decreased plasma MP production [193]. Incubation with citicoline, a membrane stabiliser, prior to TNF stimulation prevents the vesiculation of MP *in vitro* and confers some protection *in vivo* when administered as adjunct therapy, in combination with artesunate [194,195].

## Conclusions

Current scepticism about the relevance and use of murine models of malaria [50] has prompted a number of responses [16,40,196,197]. Some researchers voiced that this scepticism may affect grant funding and research publication that utilise experimental models [6], thus impinging on the progress of our understanding on this disease. Others called to facilitate better communication between scientists who utilise human studies and those that use experimental studies, sometimes involving the same people [6,13]. A number of recommendations have been made to improve translation of experimental data into clinical studies including the standardisation of models across all species and the creation of human tissue biobanks [13].

Appreciating the value of these models, and acknowledging their limitations would facilitate better research approaches to close the gap on CM. There are similarities between ECM and HCM to justify their use, although caution in the interpretation and application of the findings is paramount. Ultimately, the driving force for the study of HCM is the patient’s welfare and this can only be upheld by realistic translation of experimental findings.
